# Adult spinal cord tissue transplantation combined with local tacrolimus sustained‐release collagen hydrogel promotes complete spinal cord injury repair

**DOI:** 10.1111/cpr.13451

**Published:** 2023-03-13

**Authors:** Xinhao Zhao, Rui Gu, Yannan Zhao, Feng Wei, Xu Gao, Yan Zhuang, Zhifeng Xiao, He Shen, Jianwu Dai

**Affiliations:** ^1^ Key Laboratory for Nano‐Bio Interface Research, Division of Nanobiomedicine, Suzhou Institute of NanoTech and NanoBionics Chinese Academy of Sciences Suzhou China; ^2^ China‐Japan Union Hospital of Jilin University Changchun China; ^3^ State Key Laboratory of Molecular Developmental Biology, Institute of Genetics and Developmental Biology Chinese Academy of Sciences Beijing China; ^4^ School of Nano‐Tech and Nano‐Bionics, University of Science and Technology of China Hefei China

## Abstract

The strategy of replacing a completely damaged spinal cord with allogenic adult spinal cord tissues (aSCs) can potentially repair complete spinal cord injury (SCI) in combination with immunosuppressive drugs, such as tacrolimus (Tac), which suppress transplant rejection and improve graft survival. However, daily systemic administration of immunosuppressive agents may cause harsh side effects. Herein, a localized, sustained Tac‐release collagen hydrogel (Col/Tac) was developed to maximize the immune regulatory efficacy but minimize the side effects of Tac after aSC transplantation in complete SCI recipients. Thoracic aSCs of rat donors were transplanted into the complete thoracic spinal cord transection rat recipients, after which Col/Tac hydrogel was implanted. The Tac‐encapsulated collagen hydrogel exhibited suitable mechanical properties and long‐term sustained Tac release behaviour. After Col/Tac hydrogel implantation in SCI rats with aSC transplantation, the recipients' survival rate significantly improved and the side effects on tissues were reduced compared with those with conventional Tac medication. Moreover, treatment with the Col/Tac hydrogel exhibited similarly reduced immune rejection levels by regulating immune responses and promoted neurogenesis compared to daily Tac injections, and thus improved functional restoration. Localized delivery of immunosuppressive agents by the Col/Tac hydrogel may be a promising strategy for overcoming immune rejection of transplants, with significant potential for clinical application in the future.

## Graphical abstract

Localized delivery of immunosuppressive agents by the Col/Tac hydrogel maximized the immune regulatory efficacy but minimized the side effects of Tac after adult spinal cord tissue transplantation in complete spinal cord injury recipients, therefore improved functional restoration, which may be a promising strategy for overcoming immune rejection, with significant potential for clinical application in future.

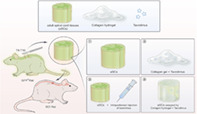

## 1 INTRODUCTION

The spinal cord functions as a bridge for signal transmission and communication between the brain and body.^1^ Trauma or disease, however, can cause spinal cord injury (SCI), which leads to cell and/or tissue death, interrupting the connections below the injured area, resulting in loss of sensation, voluntary motor dysfunction and even paralysis. Currently, SCI is still incurable.^2^ Despite the existence of strategies involving stem‐cell transplantation with or without biomaterials that could partially replace damaged cells and tissues, thereby recovering some function in SCI animals, limitations remain.^3^, ^4^, ^5^ For instance, extracellular matrix with complex structures and components, and various types of neural and nonneural cells of the spinal cord tissue are difficult to replace or restore.

In previous studies, transplantation of allogenic adult spinal cord tissues (aSCs) promoted locomotor function recovery in complete SCI rats and canines.^6^, ^7^ aSC has a natural tissue architecture and microenvironment that may be a potential substrate for replacing damaged neurons and glia and supporting axonal regrowth.^8^, ^9^ During aSC transplantation, graft survival at the lesion site is critical for successful SCI repair. Thus, systemic administration of immunosuppressive drugs has been clinically applied to avoid graft rejection‐induced apoptosis. Tacrolimus (Tac, FK506) is a clinical immunosuppressive drug that efficiently inhibits T‐lymphocyte proliferation and activation by binding to FK506‐binding protein 12 (FKBP12) to block calcineurin activation within lymphocytes.^10^, ^11^ However, severe complications, including infection, chronic allograft nephropathy, neurotoxicity, liver damage and even tumour malignancy, may occur with continuous systemic Tac administration.^12^, ^13^, ^14^

Compared with systemic administration, local drug delivery systems offer therapeutic levels of Tac at the transplantation area via sustained release for a prolonged period, which reduces toxic effects by decreasing the total drug dosage.^15^, ^16^, ^17^ Deng et al. developed poly (lactide‐co‐glycolide) nanoparticles for Tac delivery and release, which exhibited longer residence time and elimination half‐life than free Tac after injection. Hence, with this treatment, acute rejection of heterotopic transplanted hearts was reduced and allograft survival time was increased in comparison with treatment with free FK506.^15^ Further, Wu et al. designed a peptide‐based hydrogel for Tac release with immune‐responsive behaviour. The supramolecule could form a hydrogel, encapsulate the drug through π–π stacking and hydrogen bonding, and release the drug by responding to T‐cell activation. Smearing this responsive hydrogel on graft surfaces after liver transplantation significantly prolonged the hosts' survival time compared to the gavage therapeutic dose of Tac.^16^ Hence, effective Tac delivery may be a useful technique for suppressing organ transplantation rejection with fewer side effects. Furthermore, Tac reportedly enhances neural regeneration after peripheral nerve injuries.^18^, ^19^ In vitro experiments using human neural stem cells (hNSCs) co‐cultured with Tac for 7 days showed that more β‐tubulin positive neural cells were observed compared with the control, indicating the potential role of Tac in neuronal differentiation.^20^

Collagen is the main structural protein in organs and is widely used in regenerative medicine because of its excellent properties, including low immunogenicity, good biocompatibility and superior biodegradability.^21^, ^22^, ^23^ Previous studies have demonstrated that collagen hydrogel can facilitate neural cell migration, growth and differentiation, and also act as a platform to support cell and functional factor (small molecules, genes and proteins) encapsulation and delivery.^24^ Clinical studies have also indicated the potential of using collagen scaffolds developed by our laboratory in various tissue regeneration.^25^, ^26^, ^27^ Additionally, in aSC transplantation rats, adding a collagen hydrogel‐containing growth factor cocktail significantly improved graft survival levels.^6^

**FIGURE 1:**
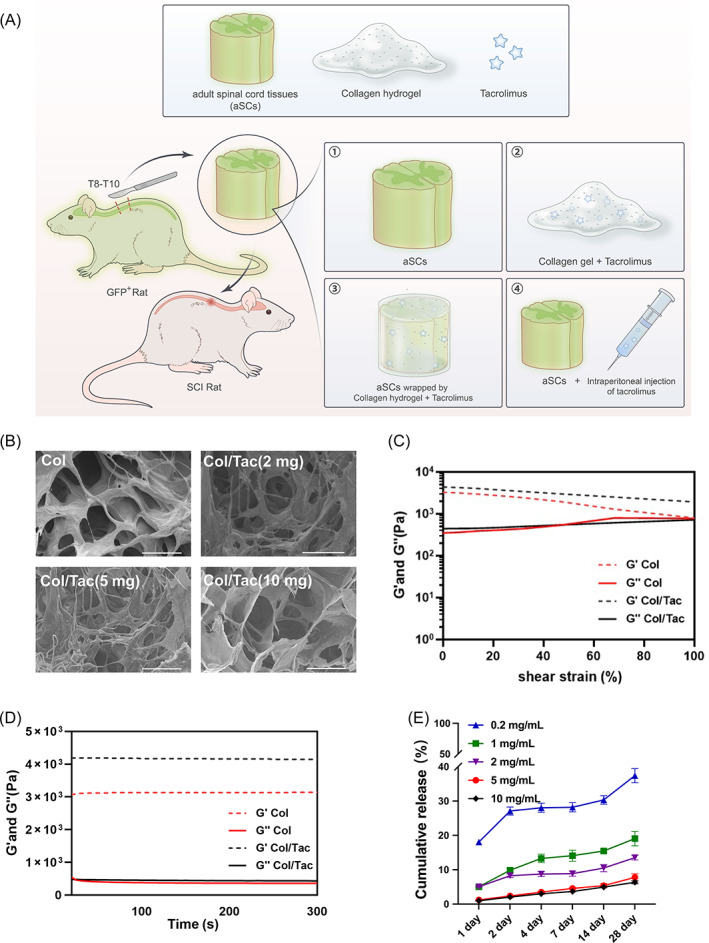
Characterization the Col/Tac hydrogel. (A) Schematic diagram of co‐transplantation of aSC and Col/Tac hydrogel for complete SCI repair. SCI rats were treated with ① aSCs, ② Col/Tac hydrogel, ③ aSC combined with Col/Tac hydrogel transplantation and ④ aSC transplantation combined with Tac intraperitoneal injection. (B) Scanning electron microscopy images of the Col/Tac hydrogel (Bar = 100 μm). (C) Shear stain curves and (D) modulus of the Col and Col/Tac hydrogel scaffolds. (E) In vitro Tac release from Col hydrogel (*n* = 3). aSC, adult spinal cord tissue; Col/Tac, collagen/tacrolimus; SCI, spinal cord injury.

In this study, we developed a collagen/Tac (Col/Tac) hydrogel to locally deliver immunosuppressive agents to improve the repair effects of the transplanted aSC by immune regulation and reduction of Tac side effects. Tac was loaded onto the collagen hydrogel (Col), and then the sustained release property was measured. The neurogenesis effects of Col/Tac were evaluated using hNSCs. Thoracic aSCs of rat donors were transplanted into the complete thoracic spinal cord transection rat recipients, after which the Col/Tac hydrogel was applied to the transplants and donor‐host interfaces to ‘glue’ the tissues together (Figure 1A). We hypothesized that the Col/Tac hydrogel could construct an immunosuppressive microenvironment after aSC transplantation to SCI recipients, consequently improving graft survival and reducing transplant rejection, thereby improving functional recovery. The effects of the Col/Tac hydrogels on regulating immune responses and promoting neural regeneration and SCI repair were studied by immunohistology analysis and transcriptomics. The side effects of the Col/Tac hydrogels were also investigated.

## 2 MATERIALS AND METHODS

### 2.1 Animals

Eighty adult female Sprague–Dawley rats (6–8 weeks old) were used as recipients, and 60 adult green fluorescent protein (GFP)‐expressing female transgenic Sprague–Dawley rats (6–8 weeks old) were used as donors. All rats were purchased from Beijing Vital River Laboratory Animal Technology Co. Ltd. (Beijing, China). Rats were housed at a standard temperature and humidity in a 12 h dark/light cycle. All animal experiments were approved by the Institutional Animal Care and Use Committee of the Chinese Academy of Sciences.

### 2.2 Surgical procedure

The surgical procedure for the T9 complete transection SCI rats was in accordance with a previous report.^28^, ^29^ All rats were anaesthetised via intraperitoneal injection with sodium pentobarbital (50 mg/kg) under aseptic conditions. A laminectomy was performed at the T9 vertebral level with an iridectomy scissor, a complete transection and removal of a 2‐mm‐long spinal cord was performed for generating hosts, and whole T8–T10 spinal cord tissues were collected from GFP‐transgenic rats. aSCs were incubated with phosphate‐buffered saline (PBS) containing 500 IU/mL heparin solution (Solarbio, China). All SCI rats were randomly divided into four groups: (1) Group aSC: aSC transplantation into lesion sites in a physiological orientation; (2) Group Col/Tac_5mg_: transplantation of 25 μL Col/Tac hydrogel with Tac concentration of 5 mg/mL at defects; (3) Group aSC + Col/Tac_5mg_: combination treatment with aSC transplantation and Col/Tac hydrogel. Briefly, the Col/Tac hydrogel was symmetrically paved at the bottom of the lesion sites and injected at the host‐graft interface, following which the spinal cord tissue surface was covered using a microsyringe (Figure S1); and (4) Group aSC + iv.Tac_d_: daily intraperitoneal Tac injection (1.5 mg/kg) after aSC transplantation. The bladders were manually emptied twice daily after surgery.

### 2.3 Preparation and characterization of the Col/Tac hydrogel

Collagen hydrogels were prepared from bovine collagen as previously described.^23^ Fresh bovine aponeurosis was treated with 1% tri(n‐butyl) phosphate for 48 h and 1% trypsin for 1 h successively, then washed with deionized water to remove cells and other proteins. The treated collagen was then dissolved in 0.5 mol L^−1^ acetic acid for 24 h, dialyzed in deionized water for 10 days, and then lyophilized. Further, 30 mg collagen powder was dissolved in 0.9 mL saline, mixed with 0.1 mL Tac solution (50 mg/mL in dimethyl sulfoxide) using a three‐way plastic tube under 4°C, and then gelatinized to form the Col/Tac hydrogel. The hydrogel morphology was observed using scanning electron microscopy (SEM, Hitachi S‐3000N, Hitachi, Tokyo, Japan) after lyophilization. Finally, the biomaterial modulus was measured using a rotational rheometer (AR2000, TA Instruments, New Castle, DE, USA).

### 2.4 Tac sustained release profile

In vitro release tests were performed using 24‐well plates. Briefly, 200 μL Col/Tac (0.2, 1, 2, 5 and 10 mg/mL) hydrogel was incubated with 400 μL PBS (pH 7.4) at 37°C, and the solution was collected at 1, 2, 4, 7, 14 and 28 days (*n* = 3). The released drug concentration was determined using high‐performance liquid chromatography tandem mass spectrometry (HPLC‐MS/MS, LC20AD‐API 3200MD TRAP, Shimadzu, Kyoto, Japan).

### 2.5 Pharmacokinetic study

To test blood concentrations of Tac, blood samples were collected from rats in all four SCI groups at 10, 30 and 60 days post‐surgery. Serum samples were collected after centrifuging the blood samples at 3000 rpm for 15 min, after which HPLC‐MS/MS analysis was performed.

### 2.6 In vivo biocompatibility test

Blood biochemistry indicators for liver, heart and kidney function were measured to assess the systemic toxicity of the Col/Tac hydrogel. The blood samples, hearts, livers, spleens, lungs and kidneys were collected. The organs were fixed in 4% paraformaldehyde, embedded in paraffin, and sectioned at 4 μm. The sections were stained with Masson and haematoxylin and eosin (H&E) and observed using an optical microscope.

### 2.7 NSC culture and differentiation

The derivation, culture and characterization of hNSCs has been described previously.^29^, ^30^ hNSCs at passage 9 were extracted from 11‐week‐old human fetal thoracic spinal cord tissues. The culture dish was coated with laminin (Sigma‐Aldrich, St. Louis, MO, USA) for hNSC adhesion. The growth medium contained 100 mg/L human plasma apo‐transferrin, 1.5% HEPES buffer solution, 25 mg/L human insulin, 20 ng/mL human LIF, 20 nM progesterone, 25 μg/L heparin, 100 μM putrescine dihydrochloride, 40 ng/mL human EGF, 40 ng/mL human bFGF, 30 nM sodium selenite, 15 g/L glucose and 2% B27 supplement in DMEM/F12. The differential medium was without EGF, bFGF, or LIF. Half medium was changed to fresh medium every 2 days.

### 2.8 Quantitative real‐time polymerase chain reaction

Total RNA samples were extracted from the NSCs after a 20‐day co‐culture with Tac, Col and Col/Tac hydrogels and converted to complementary DNA via the Invitrogen SuperScript IV First‐Strand Synthesis System (Thermo Fisher Scientific Inc., Waltham, MA, USA). Subsequently, quantitative real‐time polymerase chain reaction (qRT‐PCR) was performed using the Hieff qPCR SYBR Green Master Mix (YIASEN Biotech Co. Ltd., Shanghai, China), following the manufacturer's instructions. Relative gene expression levels of hNSCs in the Col‐only group were set to one. GAPDH was used as an endogenous housekeeping gene to normalize gene expression values. The expression levels of nestin, tubulin beta‐III (Tuj‐1), ISL LIM Homeobox 1 (Islet1), microtubule‐associated protein 2 (Map2), glial fibrillary acidic protein (GFAP) and platelet‐derived growth factor (PDGF) in the NSC samples were measured. The primer sequences used are listed in Table S1.

### 2.9 Electrophysiology

Eight weeks post‐surgery, the cortical motor‐evoked potentials (MEPs) were measured using the Keypoint bichannel‐evoked potential/electro‐myography system (9033A07, Dantec Company, Copenhagen, Denmark) as previously described.^31^ All rats received the same intraperitoneal administration of anaesthesia.

### 2.10 Behavioural assessment

The Basso–Beattie–Bresnahan (BBB) locomotor rating scale^28^ was recorded weekly by two independent observers who were unaware of the experimental conditions. The main characteristics observed were ankle movement of the hind limbs, state of feet during walking and body stability.

### 2.11 Histological analysis and immunohistochemistry

After treatment for 10 and 60 days, the animals were anaesthetised and perfused with 4% paraformaldehyde. Subsequently, 4 cm‐long spinal cord samples were collected, immersed in 30% sucrose, and embedded in a Tissue‐Tek® O.C.T compound (Sakura Finetek, Torrance, CA, USA). Further, 15 μm sections were made using a Leica CM1950 cryostat (Leica Microsystems, Wetzlar, Germany). Immunohistochemistry was performed as described previously.^28^, ^29^ Images were obtained using a Leica SP8 confocal microscope (Leica Microsystems).

The following primary antibodies were incubated overnight at 4°C: rabbit anti‐GFAP (ab7260, Abcam) 1:500, mouse anti‐Tuj‐1 (MAB5564, Millipore) 1:500, chicken anti‐GFP (ab13970, Abcam) 1:500, rabbit anti‐CD4 (MT310, Santa Cruz Biotechnology, Dallas, TX, USA) 1:500, rabbit anti‐CD8 (ab237709, Abcam) 1:500 and rabbit anti‐CD68 (ab125212, Abcam) 1:500. Fluorescent secondary antibodies from different host sources were labelled with Alexa Fluor 488, 568, or 647 (all 1:500, Invitrogen) and incubated with the samples for 1 h at 25°C.

### 2.12 RNA‐sequencing

RNA‐Sequencing (RNA‐Seq) was performed to identify the differential genes and their related pathways between the aSC + Col/Tac_5mg_ and aSC groups, and between the aSC + Col/Tac_5mg_ and aSC + iv.Tac_d_ groups, respectively. Three different batches of spinal cord samples were collected at 10 days post‐surgery and extracted their total RNA. RNA library sequencing was performed on the Illumina novaseq6000 by CapitalBio Technology Co., Ltd (Beijing, China). Bioinformatic analysis was performed using R/R‐studio and KOBAS.

### 2.13 Statistical analyses

All data were expressed as mean ± standard deviation. Statistical analyses were performed using analysis of variance test, and *p* < 0.05 was considered statistically significant. All statistical analyses were performed using SPSS (26.0, IBM, Armonk, NY, USA) and GraphPad Prism (8.0.2, Inc., San Diego, CA, USA) software packages.

## 3 RESULTS

### 3.1 Characterization and release process of the Col/Tac hydrogel

The collagen scaffold formed a white pasty hydrogel (Figure S2). SEM images of the freeze‐dried collagen hydrogel loaded with 0, 2, 5 and 10 mg/mL Tac showed porous structure (Figure 1B). The rheological characteristics were performed to study the mechanical properties of the Col and Col/Tac hydrogels. With shear strain rising from 1% to 100%, the storage modulus (G′) of the Col and Col/Tac hydrogels were decreased from 3250 and 4370 Pa to 796 and 1930 Pa, respectively, while the loss modulus (G″) were increased from 330 and 441 Pa to 795 and 723 Pa, respectively (Figure 1C). The G′ and G″ of the Col hydrogel were approximately 3300 and 400 Pa and the G′ and G″ of the Col/Tac hydrogel were about 4200 and 500 Pa after high frequency (10 Hz) scanning respectively (Figure 1D). Encapsulation of Tac increased the modulus and improved the stability of the Col hydrogel. The in vitro release properties of Tac from the Col hydrogels were investigated after incubation in HBSS after 1, 2, 4, 7, 14 and 28 days. Tac was slowly released into the medium in a time‐dependent manner in all Col/Tac hydrogels with Tac‐encapsulated concentrations of 0.2, 1, 2, 5 and 10 mg/mL (Figure 1E). Less than 10% of the Tac was rapidly released in the initial 2 days (except 0.2 mg/mL group), and approximately 15% of the drug was released within the following 26 days in a sustained manner. Finally, there was 92.2% residual Tac in the 5 mg/mL group at the 28‐day time point. Tac was mainly released because of diffusion and hydrogel degradation.

### 3.2 Effects of the Col/Tac hydrogel on hNSC differentiation

The in vitro toxicity of the Col/Tac hydrogels with different Tac concentrations (0, 1, 2, 5 and 10 mg/mL) was investigated using live/dead cell staining after culturing with hscNSCs (Figure S3A) for 1, 3 and 7 days. The Col/Tac hydrogels with Tac concentrations ≤5 mg/mL showed good biosafety with cell viabilities of 85%, which was not significantly different when compared with the cell viability of the control group (drug‐free medium) (Figure S3B–D).

**FIGURE 2:**
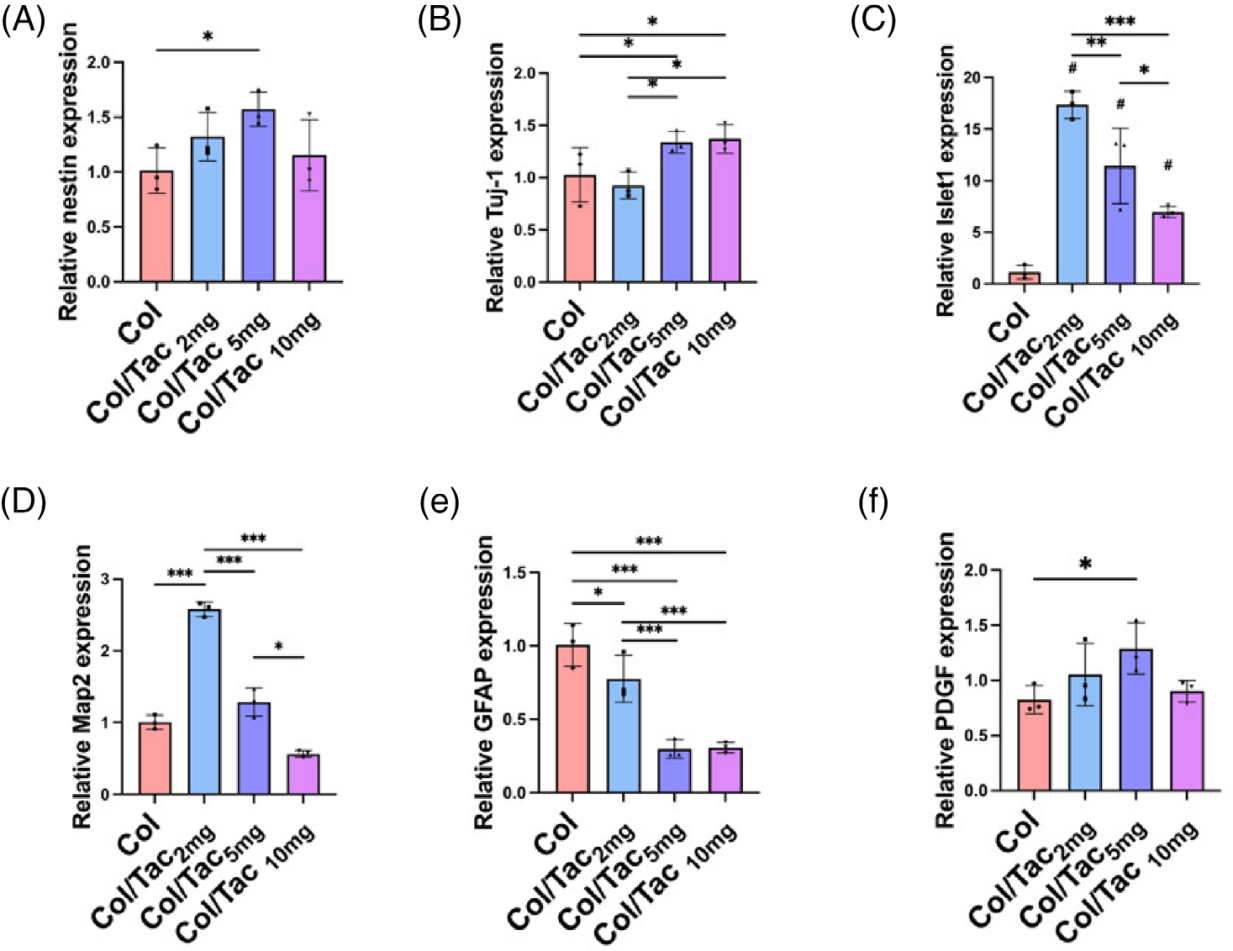
Effects of the Col/Tac hydrogel on hscNSC differentiation. Gene expression levels of (A) Nestin, (B) *Tuj‐1*, (C) *Islet1*, (D) *Map2*, (E) *GFAP* and (F) *PDGF* in the hscNSCs stimulated with the Col/Tac hydrogels loading with different Tac concentrations (0, 2, 5 and 10 mg/mL). *p < 0.05; **p < 0.01; ***p < 0.001, *n* = 3. Col/Tac, collagen/tacrolimus; hscNSC, human spinal cord‐derived neural stem cell.

The effects of the Col/Tac hydrogel on human spinal cord‐derived neural stem cell (hscNSC) differentiation were measured by the marker gene expression levels after a 20‐day co‐cultivation of hscNSCs and Col/Tac hydrogels (containing 0, 2, 5 and 10 mg/mL Tac) in neural differential medium. Nestin, a widely used marker for NSCs, was used to identify the stemness.^32^, ^33^ Nestin expression levels were higher in the Col/Tac_5mg_ group than in the Col group (Figure 2A). The expression level of *Tuj‐1*, a neuron‐specific marker, was increased in both Col/Tac_5mg_ and Col/Tac_10mg_ groups (Figure 2B). *Islet1*, a motor neuron‐related gene, showed significantly high expression in the Col/Tac_2mg_, Col/Tac_5mg_ and Col/Tac_10mg_ groups. *Map2*, a neuronal dendritic marker, was overexpressed in the Col/Tac_2mg_ group and showed slightly high expression levels in the Col/Tac_5mg_ group (Figure 2D). Moreover, the marker for astrocyte GFAP showed its highest expression level in the Col group (Figure 2E). Finally, *PDGF* expression was enhanced in the Col/Tac_5mg_ group, which is related to vessel regeneration. The gene expression behaviors in the Col/Tac_10mg_ group may have also been influenced by cell quantity as higher Tac concentrations led to cell death. These results indicated that the Col/Tac hydrogels containing appropriate Tac concentrations maintained hscNSC properties and promoted neural differentiation in vitro.

### 3.3 Pharmacokinetic analysis of Tac after Col/Tac hydrogel implantation

**FIGURE 3:**
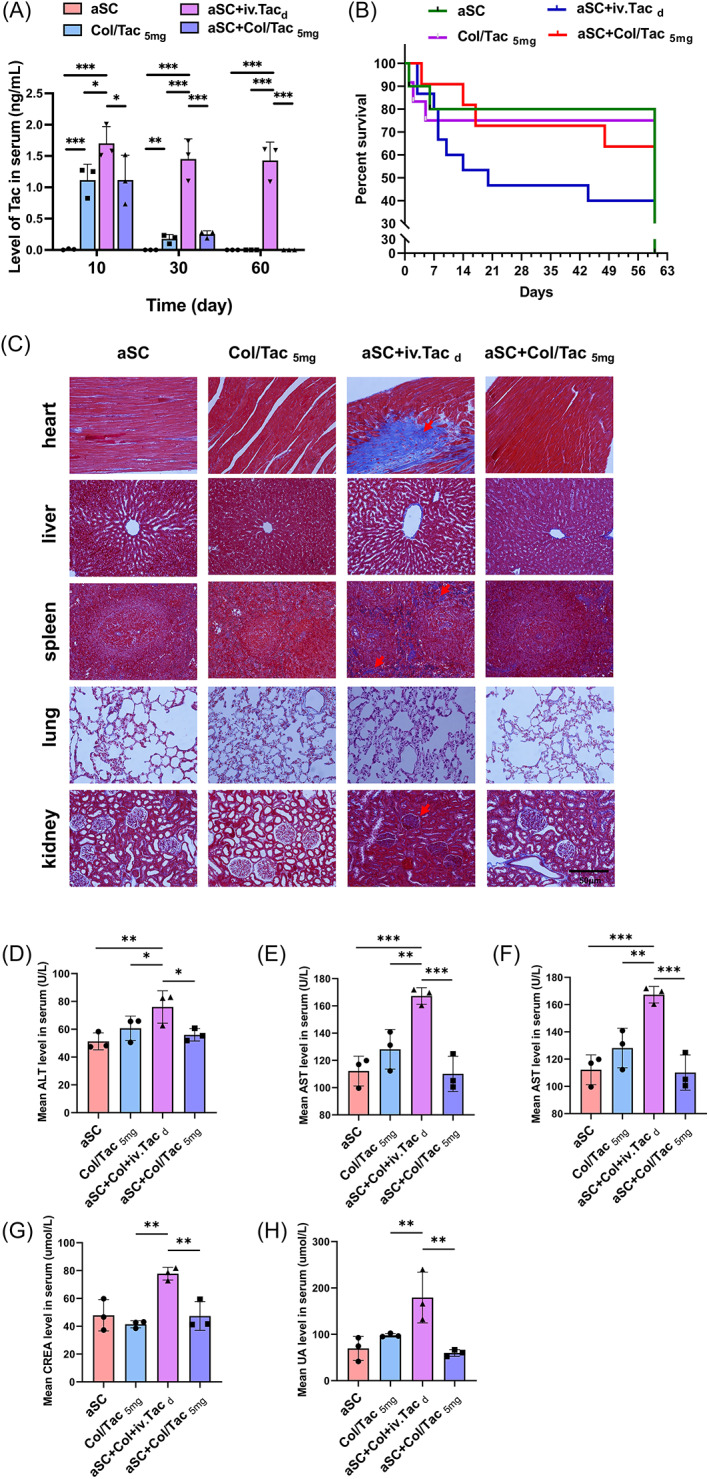
In vivo toxicity evaluation of the Col/Tac hydrogel‐treated SCI rats after aSC implantation. (A) Tac concentrations in serum in the aSC, Col/Tac_5mg_, aSC + Col/Tac_5mg_ and aSC + iv.Tac_d_ groups at different time points. Data are presented as mean ± SD (*n* = 4). (B) Survival curves of SCI rats with aSC transplantation. (C) Masson staining of the major organs. The abnormal morphologies in kidney, heart and spleen are shown with red arrows. The levels of (D) ALT, (E) AST, (F) CK, (G) CRE and (H) UA in the serum. There were no significant abnormal changes in the aSC, Col/Tac_5mg_ and aSC + Col/Tac_5mg_ groups, while noteworthy changes were observed in the aSC + iv.Tac_d_ group for kidney, heart, spleen and liver biochemistry results. Bar = 50 μm. *p < 0.05; **p < 0.01; ***p < 0.001, *n* = 3. ALT, alanine aminotransferase; aSC, adult spinal cord tissue; AST, aspartate aminotransferase; CK, creatine kinase; CRE, creatinine; Col/Tac, collagen/tacrolimus; SCI, spinal cord injury; UA, uric acid.

Tac pharmacokinetics after Col/Tac_5mg_ hydrogel implantation is crucial for immunoregulation. We evaluated Tac concentrations in blood using an HPLC/MS system at specific time points (Figure 3A). Serum Tac concentration was maintained at >1.5 ng/mL in the aSC + iv.Tac_d_ group at all time points. The Col/Tac_5mg_ and aSC + Col/Tac_5mg_ groups maintained relatively sustained and effective Tac concentrations of 1.11 ± 0.25 and 1.12 ± 0.39 ng/mL, respectively, at 10‐day post‐surgery, which decreased to 0.18 ± 0.07 and 0.25 ± 0.05 ng/mL, respectively, at 30 days. There were relatively few serum Tac residues at 60 days in the Col/Tac hydrogel implantation groups.

### 3.4 Biocompatibility evaluation of the Col/Tac hydrogels

Long‐term persistence with Tac injections is an important causal factor for tissue damage and mortality following organ transplantation.^34^ There were significant differences in the survival rates among the aSC, Col/Tac_5mg_, aSC + Col/Tac_5mg_ and aSC + iv.Tac_d_ groups during the first 30‐day post‐surgery (80%, 75%, 72% and 46.7%, respectively) and 8 weeks post‐surgery (80%, 75%, 64% and 40%, respectively). The Col/Tac hydrogel treatment enhanced the survival of aSC‐transplanted SCI rats compared to the conventional Tac treatment (Figure 3B).

Then, we performed histological tests of major organs and blood biochemistry analyses to assess the in vivo toxicity of the implantation of aSCs and Col/Tac hydrogels (containing 5 mg/mL Tac) in SCI rats at 60‐day post‐surgery. H&E staining results showed that glomerulonephritis pathomorphism was observed in the glomeruli in the aSC + iv.Tac_d_ group as they had narrow glomerular capsule gaps and glomerular basement membrane incrassation, which did not occur in the other three groups (Figure S4). This was consistent with the Masson staining results of the kidney samples in the aSC + iv.Tac_d_ group (Figure 3C). There were also large areas of abnormal fibrous connective tissues in the Masson staining results of the heart and spleen in the aSC + iv.Tac_d_ group, which may be associated with hypofunction. Furthermore, alanine aminotransferase (ALT) and aspartate aminotransferase (AST) were chosen as biochemical indicators for assessing liver toxicity, creatine kinase (CK) for cardiac toxicity, and uric acid (UA) and serum creatinine (CRE) for kidney toxicity (Figure 3D–H). Significantly higher serum levels of the above metabolites were detected in the aSC + iv.Tac_d_ group compared with the others. The results showed that the Col/Tac localized delivery and sustained release hydrogel, when combined with aSC transplantation, did not result in significant toxicity 60‐day post‐surgery, while the aSC + iv.Tac_d_ treatment affected the heart, liver and kidney function. The local Tac sustained‐release hydrogel caused lower toxicity levels than the daily Tac injection method.

### 3.5 Effects of the Col/Tac hydrogel on peripheral immune cells after aSC transplantation

**FIGURE 4:**
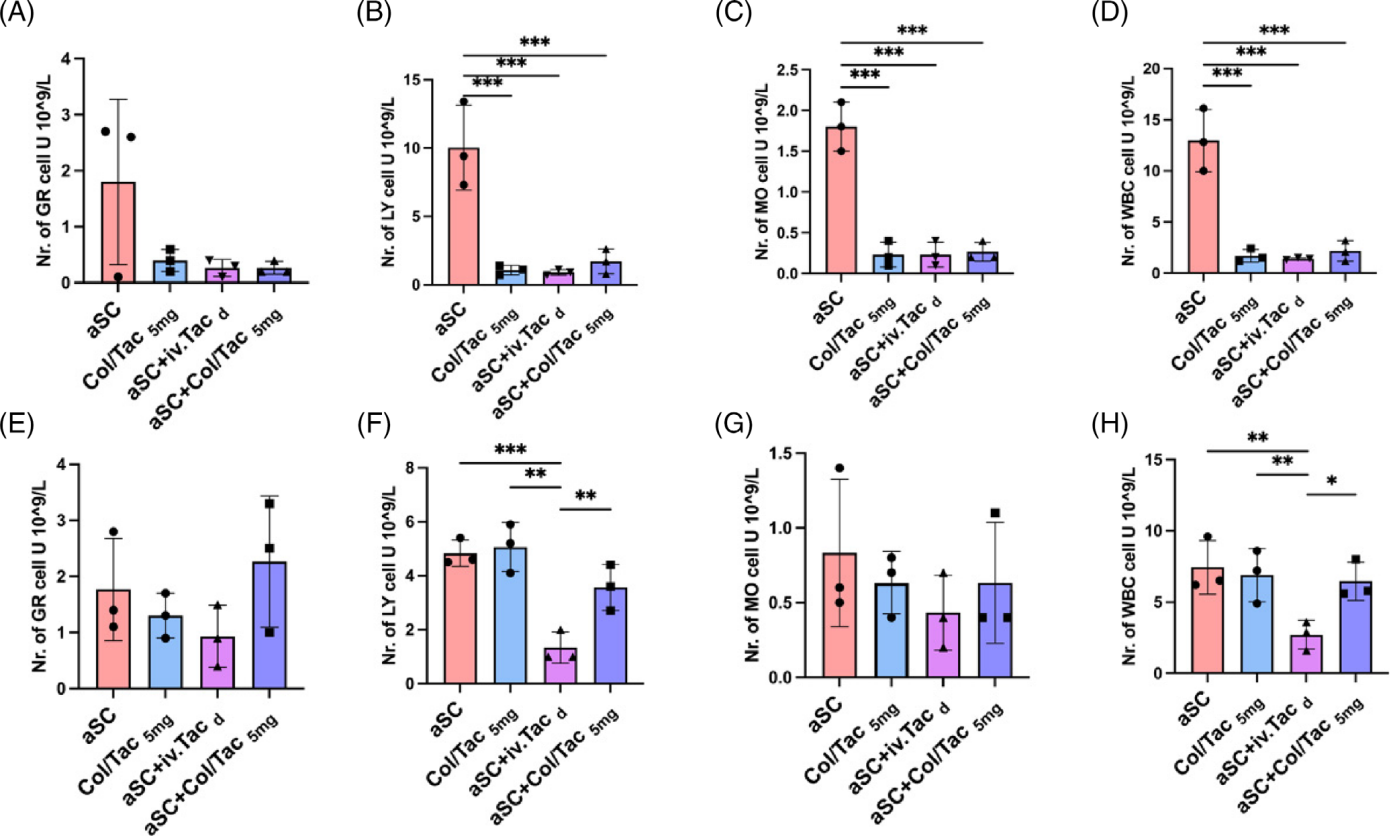
Levels of peripheral immune cells in SCI rats with aSC transplantation. Counts of (A, E) granulocytes (GR), (B, F) lymphocytes (LY), (C, G) monocytes (MO) and (D, H) total white blood cells (WBCs) in the peripheral blood at 10 days (A–D) and 60 days (E–H) post‐transplantation. *p < 0.05; **p < 0.01; ***p < 0.001, *n* = 3. aSC, adult spinal cord tissue; SCI, spinal cord injury.

Peripheral immune cell counts can reflect the host's systemic immune status. We obtained peripheral white blood cell (WBC) counts including counts of granulocytes (GR), lymphocytes (LY), monocytes (MO) and total WBCs at 10 and 60‐day post‐injury. With both daily Tac administration and localized Col/Tac hydrogel implantation, peripheral immune cell counts were reduced during the acute rejection period (10‐day post‐transplantation; Figure 4A–D). In particular, LY, MO and total WBC counts were significantly reduced when compared with those in the aSC group (*p* < 0.001), and no significant difference was found between the aSC + Col/Tac_5mg_ and aSC + iv.Tac_d_ groups at subacute stage. For 60‐day post‐transplantation, LY and WBC counts were persistently lower in the aSC + iv.Tac_d_ group than in the aSC, Col/Tac_5mg_ and aSC + Col/Tac_5mg_ groups (Figure 4E–H). The local Tac sustained‐release hydrogel showed similar effects with daily Tac administration on peripheral immune cells regulation at acute rejection periods, but could not maintain in long‐term.

### 3.6 Col/Tac hydrogel attenuated immune rejection and inflammation at lesion site

**FIGURE 5:**
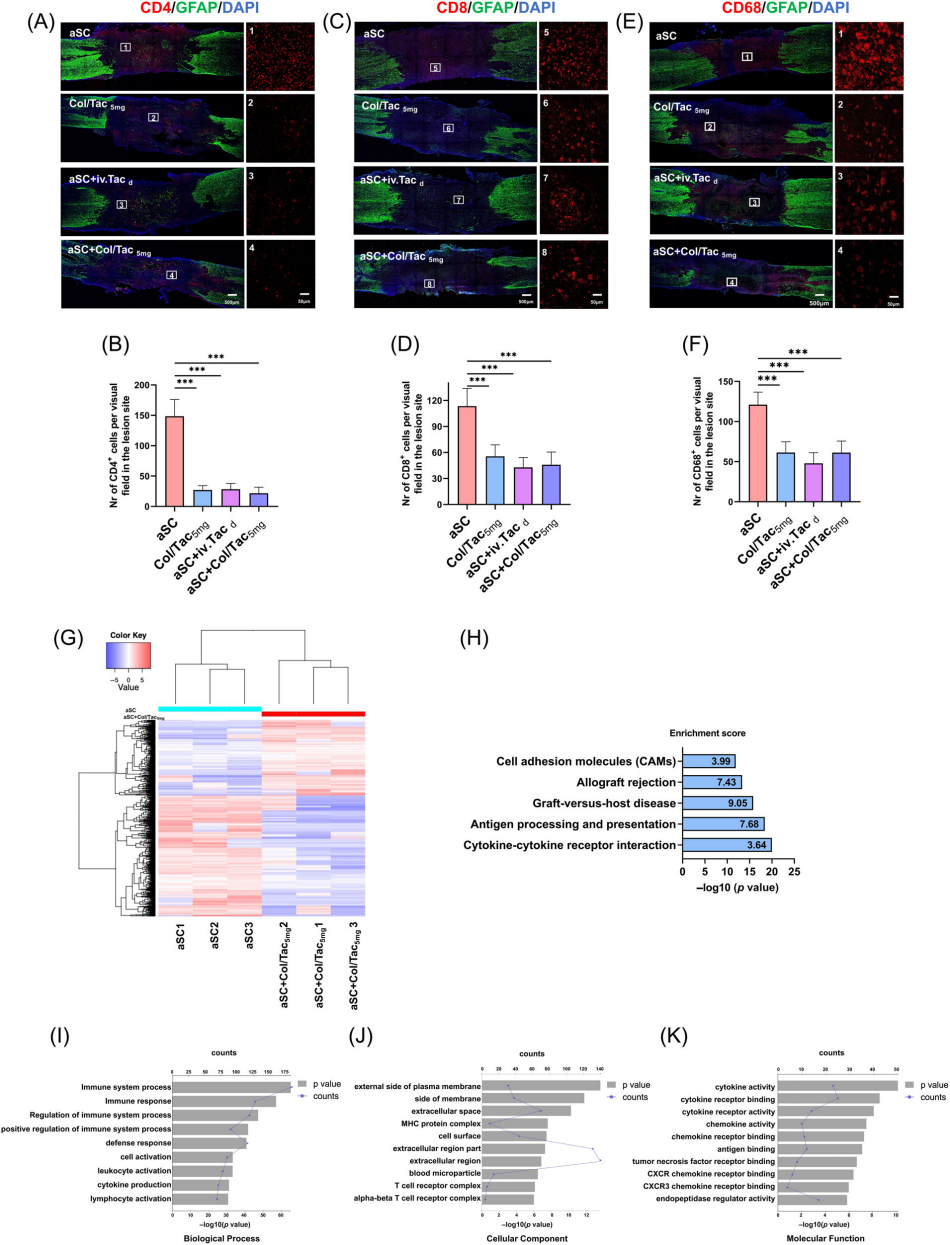
Immune rejection and inflammatory responses in the transplant area. Immunostaining images and semi‐quantification of (A, B) CD4+ T cells, (C, D) CD8+ T cells and (E, F) CD68+ macrophage/microglia cells in the transplant area at 10‐day post‐transplantation. Bar = 250 μm. ***p < 0.001, *n* = 5. (G) Cluster analysis of differentially expressed gene comparing the aSC + Col/Tac_5mg_ group with the aSC group. (H) Graph showing KEGG enrichment pathways compared with the aSC + Col/Tac_5mg_ and aSC groups. The top 10 pathways of GO analysis of the differentially expressed genes between the aSC + Col/Tac_5mg_ and aSC groups based on (I) biological process, (J) cellular component and (K) molecular function.

Severe and rapid reactive immune rejection is the main cause for cell and organ transplantation failure.^35^, ^36^ Cellular immunity mediated by T cells, including regulatory T cells (CD4+) and cytotoxic T cells (CD8+), plays important roles in this process.^37^ Regulatory T cells can recognize and present antigens and secrete cytokines, including IL‐2, IFN‐γ and TNF.^15^ Cytotoxic T cells can directly induce cell apoptosis and death.^38^ To investigate immune rejection after aSC transplantation, immunofluorescence staining of CD4 and CD8 was performed for T cells at 10‐day post‐transplantation. Fewer CD4+ and CD8+ T cells were observed in the Col/Tac_5mg_, aSC + Col/Tac_5mg_ and aSC + iv.Tac_d_ groups compared to the aSC group (Figure 5A,C). The number of CD4+ and CD8+ T cells was not visibly different among the Col/Tac_5mg_, aSC + Col/Tac_5mg_ and aSC + iv.Tac_d_ groups (Figure 5B,D). These data indicated that localized Tac delivery via Col/Tac hydrogel exhibited similar immunoregulatory effects to the daily Tac administration during subacute phase, which reduced local immune rejection.

The activated macrophage‐ and microglia‐mediated inflammatory microenvironment impairs neurological recovery after SCI.^39^ Immunofluorescence staining of CD68, a marker of reactive microglia/macrophages, was used to determine the inflammatory response in the transplant area 10‐day post‐surgery (Figure 5E). The number of CD68‐positive cells was decreased in the Col/Tac_5mg_, aSC + Col/Tac_5mg_ and aSC + iv.Tac_d_ groups when compared with those in the aSC group (Figure 5F). The staining results of CD68, CD4 and CD8 indicated that an immunosuppressive microenvironment at the lesion site was constructed by Col/Tac hydrogel implantation, which might be beneficial for graft survival and neural restoration.

RNA‐Seq analysis at day 10 of aSC transplantation showed that 711 genes exhibited significant differential expression levels with 33.3% upregulation and 66.7% downregulation after Col/Tac hydrogel implantation (*p* < 0.05, log_2_ Fold Change > 1; Figure 5G). Kyoto Encyclopedia of Genes and Genomes (KEGG) pathway enrichment analysis revealed that these differentially expressed genes (DEGs) were mainly in the pathways related to graft‐versus‐host disease, antigen processing and presentation, allograft rejection, cytokine receptor interaction and cell adhesion molecules (Figure 5H). Biological process, cellular component and molecular function gene ontology (GO) analysis showed the top 10 pathways involved in immune responses, membrane function and cytokine activation (Figure 5I–K). These results suggested that implanting Col/Tac after aSC transplantation mainly regulated immune cell migration, cytokine production and immune cell activation. Furthermore, KEGG analysis revealed that the DEGs between the localized sustain Tac delivery and conventional Tac medication were mainly enriched in chemokine signalling pathway, neuroactive‐ligand receptor interaction and axon guidance categories, rather than immune response‐related categories (Figures S5 and S6). The results of the GO enrichment analysis showed that DEGs between the aSC + Col/Tac_5mg_ and aSC + iv.Tac_d_ groups were primarily involved in chemokine‐related pathways and extracellular region (Figure S7).

### 3.7 Implantation of Col/Tac hydrogel improves the transplanted aSC survival

**FIGURE 6:**
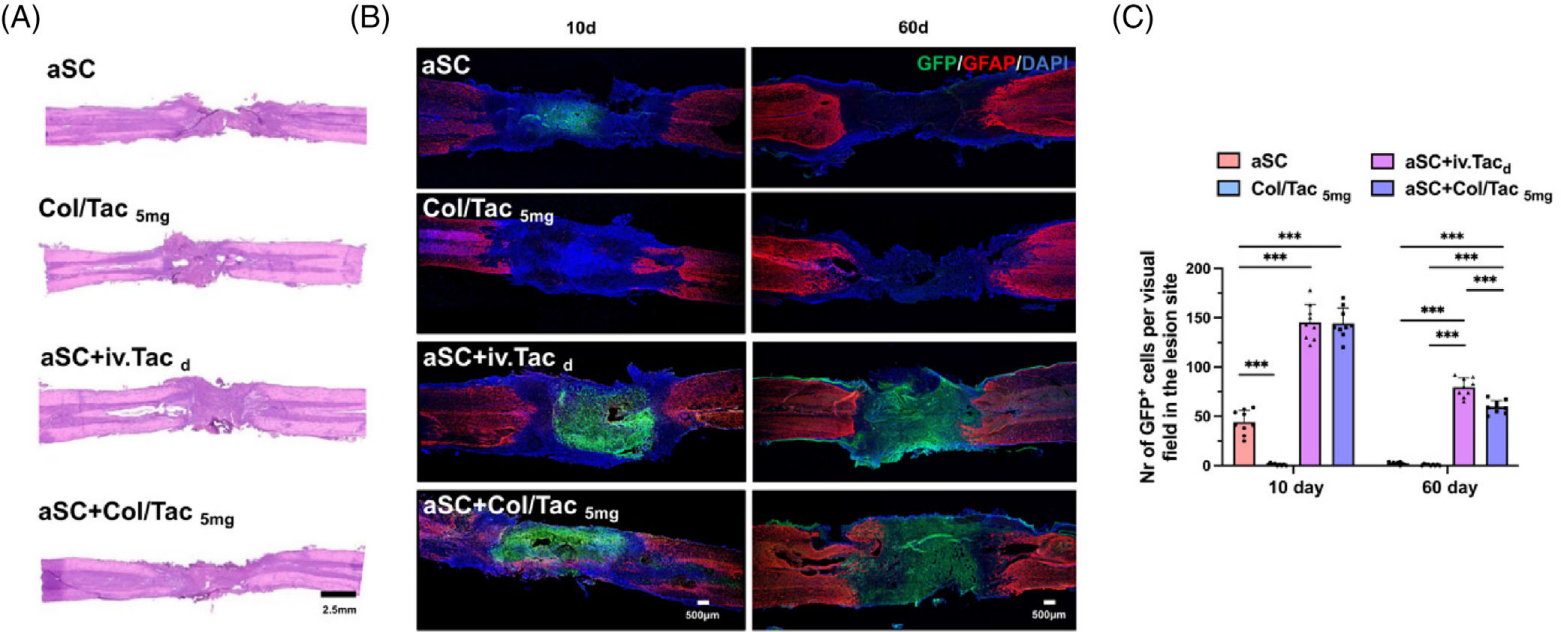
Transplanted aSC survival at lesion sites. (A) Representative haematoxylin and eosin staining images of each group at 60‐day post‐surgery. Bar = 2.5 mm. GFP (green) represents the donor segment cells. GFP immunostaining images (B) and quantification (C) of GFP‐positive cells in the lesion areas at 10 days and 60 days post‐transplantation. Bar = 500 μm. ***p < 0.001, *n* = 5. aSC, adult spinal cord tissue; GFP, green fluorescent protein.

A previous report evidenced that aSC transplantation could improve locomotor function recovery in SCI rats.^6^ aSC survival is the basis for this remedy. To investigate the transplanted aSC integration status in the host, the formation of cystic cavities and tissue necrosis was evaluated in the spinal cord samples of the SCI rats using H&E staining at 60‐day post‐surgery (Figure 6A; Figure S8). No significant tissue necrosis was observed. Moreover, immunofluorescence staining with GFP identified numerous donor cells in the transplanted area of the aSC + Col/Tac_5mg_ and aSC + iv.Tac_d_ groups at 10‐ and 60‐day post‐surgery compared to those in the aSC group (Figure 6). GFP+ donor cell counts in the aSC + Col/Tac_5mg_ group showed similar levels to those in the aSC + iv.Tac_d_ group at 10‐ and 60‐day post‐transplantation. This suggested that the Col/Tac hydrogel treatment significantly enhanced the survival levels of the transplanted aSCs, possibly contributing to function restoration (Figure 6C).

**FIGURE 7:**
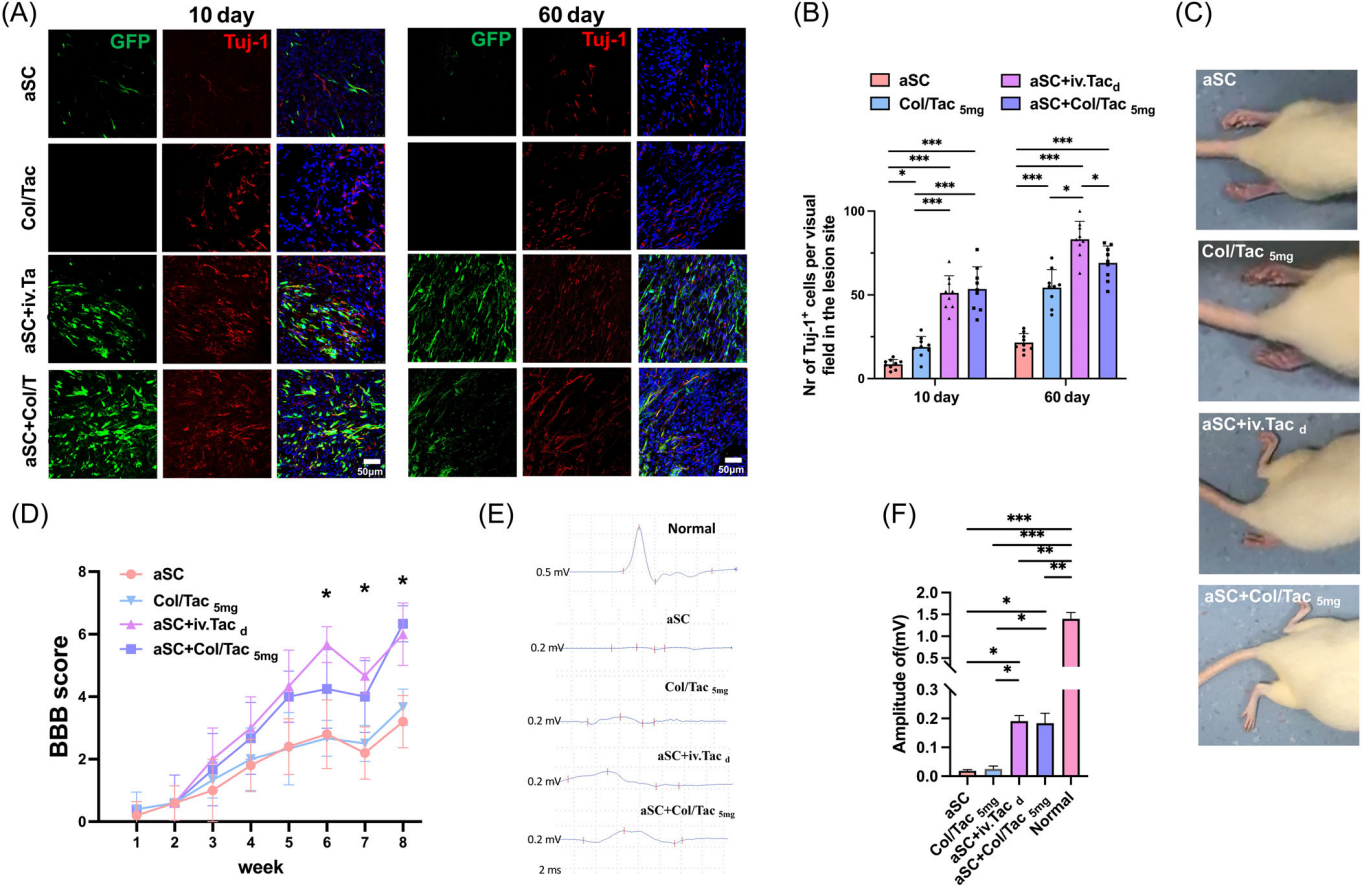
(A) Immunostaining images of Tuj‐1‐positive neurons (red) and GFP‐positive cells (green) in the transplanted aSCs of the spinal cord samples 10‐ and 60‐day post‐injury. (B) Semi‐quantification of Tuj‐1‐positive cells in the lesion areas at 10‐ and 60‐day post‐transplantation. (C) Typical images of the feet of SCI rats after treatment for 60 days. (D) BBB scores for the rats' hind limbs. (E) Motor evoked potential (MEP) and (F) MEP amplitude for the SCI rats in each group 60‐day post‐surgery. Bar = 50 μm, *p < 0.05; **p < 0.01; ***p < 0.001, *n* = 5. aSC, adult spinal cord tissue; BBB, Basso–Beattie–Bresnahan; GFP, green fluorescent protein; SCI, spinal cord injury.

We further evaluated neuron survival in the transplanted site by immunostaining of neuron‐specific Tuj‐1 and GFP (Figure 7A). Many GFP‐ and Tuj‐1‐co‐labelled neurons were detected in the aSC + Col/Tac_5mg_ and aSC + iv.Tac_d_ groups 10‐ and 60‐day post‐surgery. Moreover, we found that the Col/Tac hydrogel implantation could only increase neuron cell numbers at defects compared to aSC transplantation (Figure 7B). This phenomenon is consistent with the above in vitro experiments. Additionally, the Col/Tac hydrogel implantation could prevent myelin degeneration of transplanted aSCs (Figure S9).

### 3.8 aSC + Col/Tac implantation improved function recovery

MEPs and BBB scores were used to evaluate motor function recovery levels after treating SCI rats with aSC and Col/Tac hydrogel. After treatment for 8 weeks, aSC and Col/Tac_5mg_ rats showed slight sweeping hind limbs, while aSC + iv.Tac_d_ and aSC + Col/Tac_5mg_ rats could frequently swing their hind limbs and take occasional weight‐supported plantar steps (Figure 7C). Mean BBB scores of the aSC‐transplanted SCI rats with Col/Tac treatment were restored to approximately 6, which were similar to those with daily Tac administration, indicating that two joints were involved in substantial movement and one in light movement; moreover, SCI rats in the aSC group had less than 4 mean BBB scores (Figure 7F). SCI rats in the aSC + Col/Tac_5mg_ and aSC + iv.Tac_d_ groups showed notable electrophysiological improvements compared to those in the aSC group (Figure 7D). The mean MEP amplitude in the aSC + Col/Tac_5mg_ and aSC + iv.Tac_d_ groups was restored to 0.18 and 0.19, respectively, which was higher than that in the aSC and Col/Tac_5mg_ groups (Figure 7E). These results suggested that the co‐implantation of aSC and Col/Tac hydrogel facilitated motor function recovery in SCI rats.

## 4 DISCUSSION

Transplantation of central neural tissues was reported as an effective strategy for replacing damaged spinal cord and reconstructing neural circuits for SCI therapy.^6^ However, immune rejection and inflammatory responses after organ transplantation result in reduced graft survival and function loss, leading to poor therapeutic efficacy. Tac is currently the drug of choice for immunosuppression, which has been commercialized in over 70 countries to date.^35^, ^40^ Tac is also the first choice for transplanting allogeneic or xenogeneic grafts for SCI therapy.^41^, ^42^ However, the toxicity and side effects caused by immunosuppressants are largely ignored.^43^ Using a Tac dose of 3 mg/kg/day could result in continued body weight decline in cell transplantation treatment for amyotrophic lateral sclerosis.^44^ There were inevitable complications, including infection, chronic allograft nephropathy, neurotoxicity and liver damage, in organ transplantation clinical research caused by Tac, further accelerating SCI complication owing to low resistance.^12^, ^13^ In our previous in vivo experiments, dramatic weight loss, lethargic behaviour, lacklustre fur and low survivorship occurred because of daily immunosuppressive injections.

New strategies for Tac administration were designed to reduce side effects and improve therapeutic efficiency.^14^, ^16^, ^45^ An immune‐responsive hydrogel was applied in a local controlled release system, which prolonged liver transplant survival.^16^ Similar release methods were created and validated in different animal models, showing great potential for cell and organ transplantations. However, the complex synthetic process and uncertain biosecurity of the high molecular synthetic material mean that it is not yet ready for clinical application.^46^ Collagen is a natural protein that occurs mainly in the extracellular matrix, with reliable biocompatibility.^47^ Our laboratory developed a series of clinical grade collagen scaffolds for various growth factor‐localized delivery and sustained‐release, and have been used to treat patients of SCI, intrauterine adhesion, so forth.^21^, ^25^, ^26^ The Col hydrogel used in this study went through an authorized third‐party inspection by the China Food and Drug Administration, and met the Chinese Criterion for Medical Devices (GB16886). Thus, the Col/Tac hydrogel presents great potential in clinical applications of organ transplantation as it has several advantages over daily Tac administration. Col/Tac implantation in the aSC‐transplanted SCI rats had similar effects on graft survival, neural regeneration and functional restoration to daily Tac treatments but had significantly lower toxicity levels.

Transplant rejection is a series of T‐lymphocyte‐mediated complex responses.^48^ In nude athymic rats, which lack a normal thymus and functional T cells, the transplanted human cells could survive for up to 6 months.^49^ Moreover, CD4+ T cells turn into helper cells in response to foreign antigens, and subsequently activate CD8+ T cells to play a cytotoxic role while activating microglia/macrophages.^50^ The results confirmed that the Col/Tac hydrogel could efficiently regulate the immune microenvironment by reducing T cells and macrophages at the transplant site, thereby reducing immune response‐mediated secondary injuries and aSC death and demyelination. Moreover, the transcriptomics results also confirmed that the Col/Tac hydrogel mainly regulated the immune rejection responses after aSC transplantation. Even though the total Tac dosage applied in the Col/Tac hydrogel implantation group was approximately 70‐fold less than in the Tac injection group during the 60‐day experiment, the immunoregulatory effects were similar due to the localized delivery and sustained release behaviour of the Col/Tac hydrogel.

Tac binds FKBP12 to form a complex that inhibits calcineurin and leads to the inhibition of T‐lymphocyte proliferation.^11^ Notably, FKBP and calcineurin are co‐expressed at high levels in most brain and spinal cord regions, indicating that they have key regulatory functions.^51^ The Tac‐FKBP12 complex increased neuronal levels of GAP‐43 protein, which is involved in neural injury response and regeneration.^52^ In contrast to cyclosporin A and rapamycin, Tac did not inhibit hNSC proliferation.^20^ When released in a locally injured peripheral nerve area, Tac enhanced axon regeneration and repaired sciatic and peroneus nervous injury.^53^ The neuroprotective effects and neurotrophy of Tac were evidenced in peripheral nerve injury.^18^, ^45^ Although the functional restoration effects of Tac administration in SCI animals were disputed,^54^, ^55^ Tac was found to drive hNSCs toward a neuronal fate in vitro, likely owing to Notch blockade.^20^ We also observed that the presence of Col/Tac enhanced neural differentiation of hNSCs. The RNA‐seq analysis also confirmed that treatment of Col/Tac in the aSC transplanted SCI rats exhibited positive effects on axon growth compared to conventional Tac daily injection. Thus, Tac may not only be an immunosuppressive agent but also a potential contributor to SCI therapy. For instance, combined treatment with Tac and nerve growth factor exhibited synergistic effects in both peripheral nerve injury and SCI.^56^ In addition, transcriptional analysis also revealed that Col/Tac implantation in the aSC transplanted SCI rats might regulate cellular development and axon compared to conventional daily Tac administration, which might exhibit positive effects on SCI repair.

Cell and tissue transplantation reportedly produced potential benefits in SCI treatments in the previous two decades; however, a lack of standardization between studies, including immunosuppression regimes, has limited development.^43^ Unlike solid organ transplantation, there is no vascular remodelling at the graft site. Therefore, effective immunosuppressant concentration in the injured spinal cord area could be lower than that in the blood. This could explain why survival results of transplanted cell and tissue are unsatisfactory, even though an immunosuppressant megadose with prominent side effects is still used post‐transplantation. To address this, we have developed a collagen hydrogel‐based tissue transplantation strategy, which could provide localized delivery and sustained release of Tac at defects. The release trend conformed to the strategy of induction and maintenance immunosuppression used in clinical organ transplantation when a megadose is applied in the acute stage and half dose in the maintenance period.^57^, ^58^

## 5 CONCLUSIONS

Col/Tac hydrogel was constructed to localize delivery and sustained release immunosuppressive agent for improving the transplanted aSC survival levels post‐implantation and reduce systemic toxicity and side effects of daily drug injection. Co‐implantation of aSC and Col/Tac hydrogel attenuated local immune rejection and promoted neural regeneration, thus enhanced therapeutic effects of the transplants and improving functional recovery in complete SCI rats. Hence, we believe that collagen hydrogel‐based immunosuppressive agent local delivery and release substrate provides a promising strategy for improving the therapeutic efficiency of cell/tissue/organ transplantation in future clinical applications.

## AUTHOR CONTRIBUTIONS

Jianwu Dai and He Shen conceived and designed the project. Xinhao Zhao performed the experiments; Yannan Zhao, Xu Gao, and Yan Zhuang contributed to data acquisition. Xinhao Zhao, Zhifeng Xiao and Yan Zhuang analysed the data; Xinhao Zhao and He Shen wrote and edited the manuscript; He Shen and Jianwu Dai contributed to the manuscript revision and supervised the research. All authors read and approved the final paper.

## FUNDING INFORMATION

This work was supported by grants from the Strategic Priority Research Program of the Chinese Academy of Sciences (No. XDA16040701), the Major Program of National Natural Science Foundation of China (No. 81891002), the National Natural Science Foundation of China (No. 81971178), the Youth Innovation Promotion Association CAS (No. 2021319), and the Jilin Provincial Science and Technology Program (No. 20200201341JC).

## CONFLICT OF INTEREST STATEMENT

The authors declare no competing interests.

## Supporting information


**Data S1.** Supporting Information

## Data Availability

The data that support the findings of this study are available from the corresponding authors upon reasonable request.
