# Supplementary material for: Adult spinal cord tissue transplantation combined with local tacrolimus sustained‐release collagen hydrogel promotes complete spinal cord injury repair
Source: Cell Prolif. 2023 Mar 13;56(5):e13451. doi: 10.1111/cpr.13451 (PMC10212717; doi:10.1111/cpr.13451)
Supplement: Supplementary file 1 — Data S1. Supporting Information [file CPR-56-e13451-s001.docx]

**Supporting information**

Adult spinal cord tissue transplantation combined with local tacrolimus sustained-release collagen hydrogel promotes complete spinal cord injury repair

Xinhao Zhao^1,2,3,^¶, Rui Gu^2,^¶, Yannan Zhao^3^, Feng Wei^1,4^, Xu Gao^1,2^, Yan Zhuang^1,4^, Zhifeng Xiao^3^, He Shen^1,4,^*, Jianwu Dai^1,3,4,^*

^1^Key Laboratory for Nano‐Bio Interface Research, Division of Nanobiomedicine, Suzhou Institute of NanoTech and NanoBionics, Chinese Academy of Sciences, Suzhou 215123, China

^2^China-Japan Union Hospital of Jilin University, Changchun, 130033, China

^3^State Key Laboratory of Molecular Developmental Biology, Institute of Genetics and Developmental Biology, Chinese Academy of Sciences, Beijing 100101, China

^4^School of Nano-Tech and Nano-Bionics, University of Science and Technology of China, Hefei 230026, China

¶ These authors contributed equally to this work.

**Correspondence:**

Jianwu Dai, Key Laboratory for Nano‐Bio Interface Research, Division of Nanobiomedicine, Suzhou Institute of NanoTech and NanoBionics, Chinese Academy of Sciences, 398 Ruoshui Road, Suzhou, Jiangsu, 215123, China. Email: jwdai@genetics.ac.cn

He Shen, Key Laboratory for Nano‐Bio Interface Research, Division of Nanobiomedicine, Suzhou Institute of NanoTech and NanoBionics, Chinese Academy of Sciences, 398 Ruoshui Road, Suzhou, Jiangsu, 215123, China. Email: [hshen2009@sinano.ac.cn](mailto:hshen2009@sinano.ac.cn)

**Table S1.** PCR primer sequences

| **Gene** | **Sense primer (5’-3’)** | **Antisense primer (5’-3’)** |
| --- | --- | --- |
| Nestin | CCA CCC TGC AAA GGG AAT CT | GGT GAG CTT GGG CAC AAA AG |
| Tuj-1 | TAG ACC CCA GCG GCA ACT AT | GTT CCA GGT TCC AAG TCC ACC |
| Islet1 | CGT GCC CGC TCC AAG GTG TAT CA | CAT TGG GCT GCT GCT GCT GGA GT |
| Map2 | ACC CTT CTA CCC CTC TCC TT | GAT CAA TAA AAT CTG CGC AG |
| GFAP | CAG TTA TCA GGA GGC GCT GG | TTT GCC CCC TCG AAT CTG CC |
| PDGF | CTG GAC ACT GGG AGA TTC GG | CAC GGC CTC CAA TGA TCT CT |
| GAPDH | ACC CAG AAG ACT GTG GAT GG | CAC ATT GGG GGT AGG AAC AC |


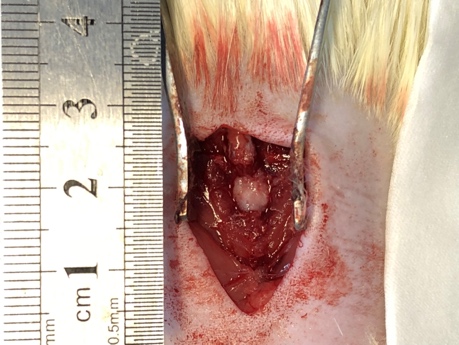


**Figure S1**. Photo of surgical procedure. Co-transplantation of aSC and the Col/Tac hydrogel to the lesion site of the complete SCI rats.


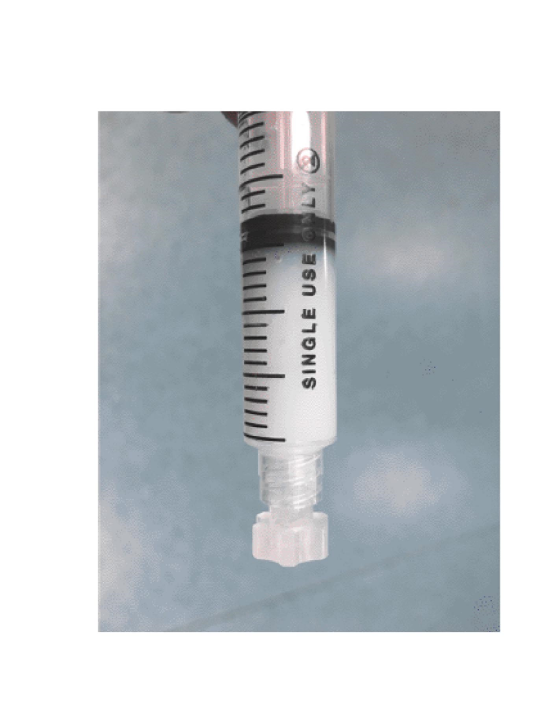


**Figure S2.** Photo of the Col hydrogel.


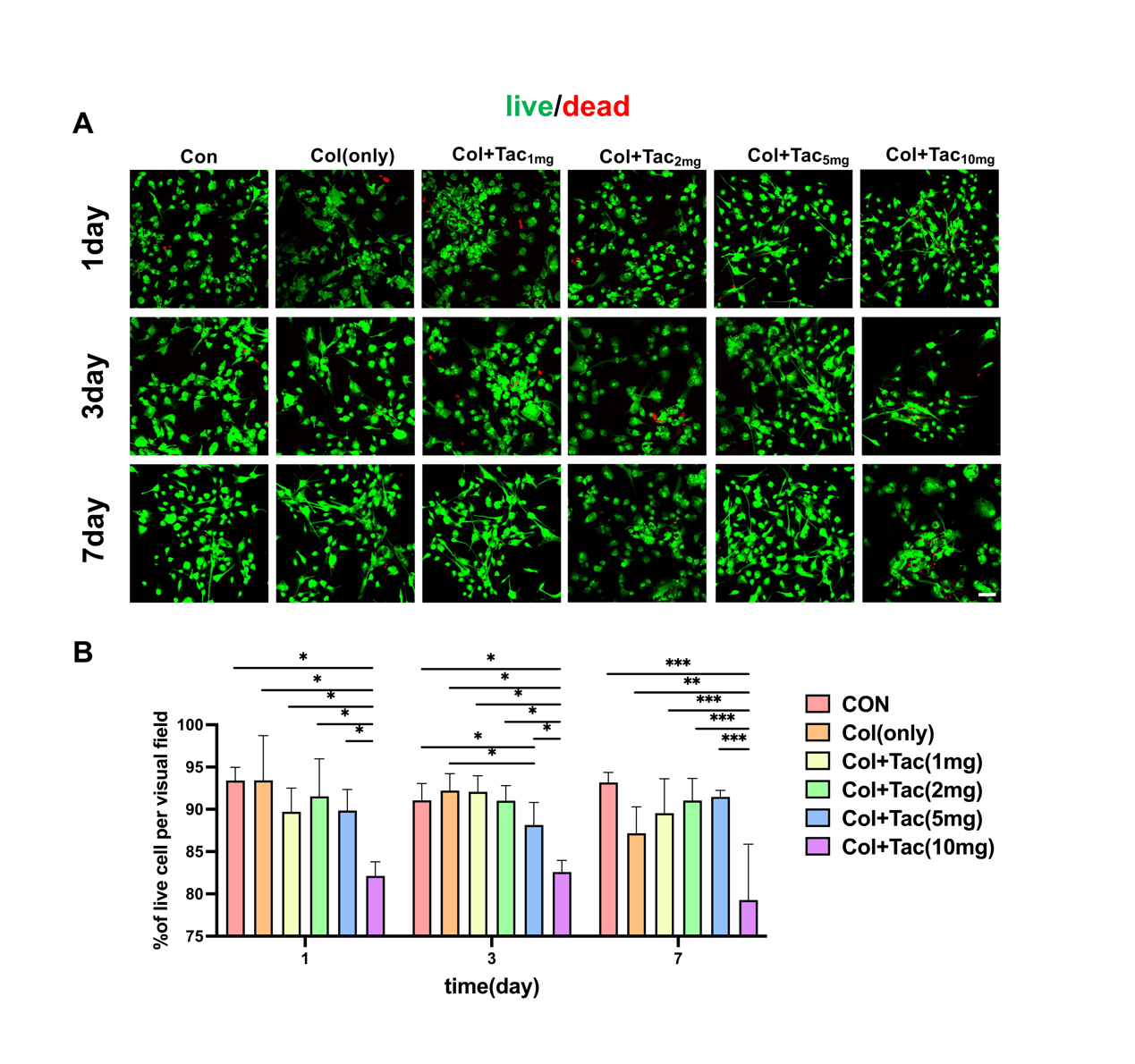


**Figure S3**. Cell viability of hscNSCs treated with the Col/Tac hydrogels. (A) Live/dead staining and (B) semi-quantification of hscNSCs incubated with different Col/Tac hydrogels (Tac concentration: 0, 1, 2, 5, and 10 mg/mL) for 1, 3, and 7 days, respectively. Scale bar = 50 μm.*p < 0.05, ***p < 0.001, n = 3.


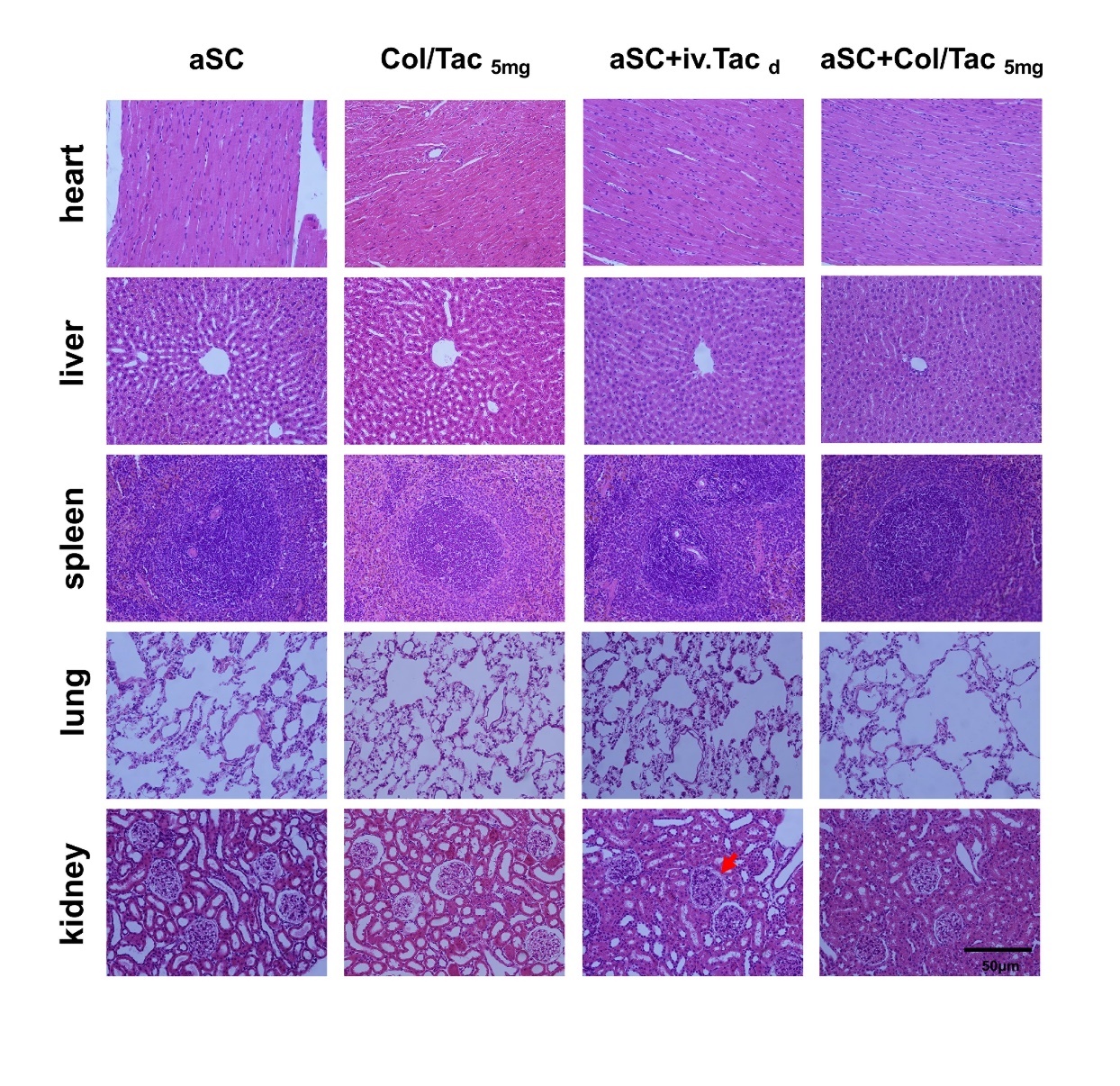


**Figure S4**. HE staining of the major organs. The abnormal morphologies in kidney, heart, and spleen are shown with red arrows.


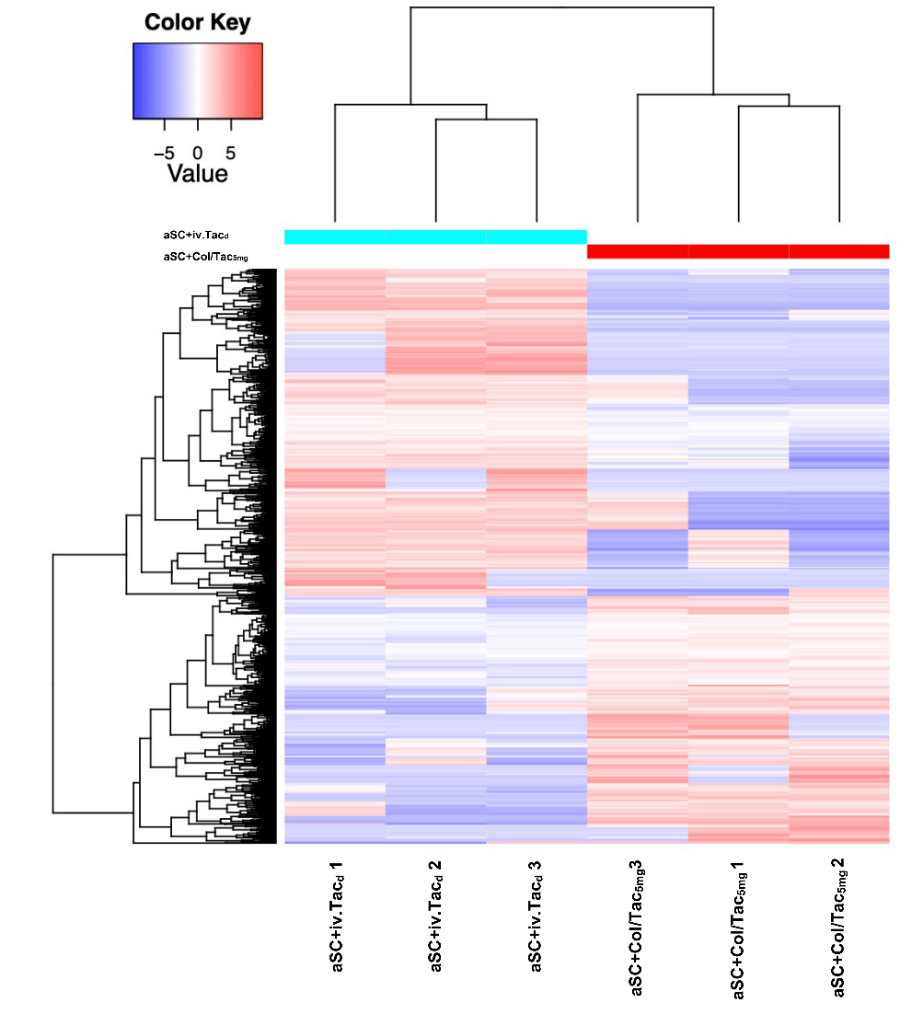


**Figure S5**. Heatmap of the differentially expressed genes between aSC+iv.Tac_d_ and aSC+Col/Tac_5mg_ groups (p < 0.05, log_2_FC > 1).


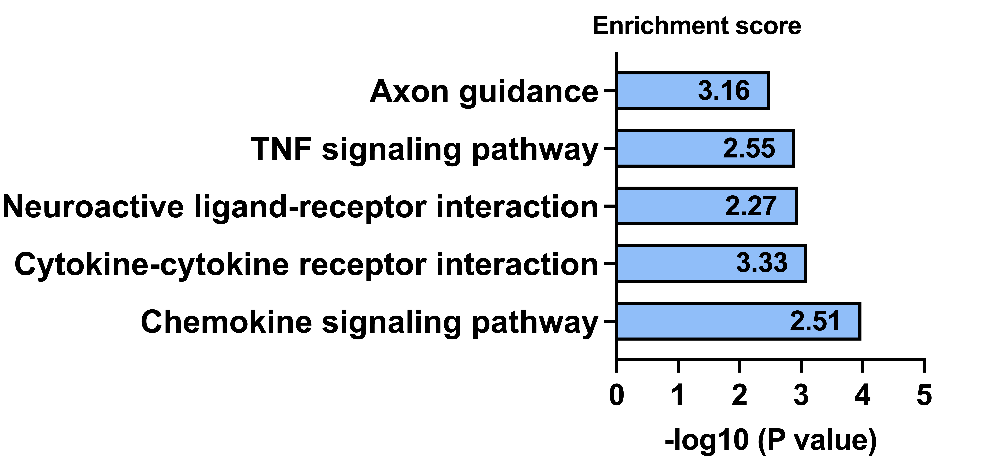


**Figure S6.** KEGG analysis of the differentially expressed genes between aSC+Col/Tac_5mg_ and aSC+iv.Tac_d_ groups.


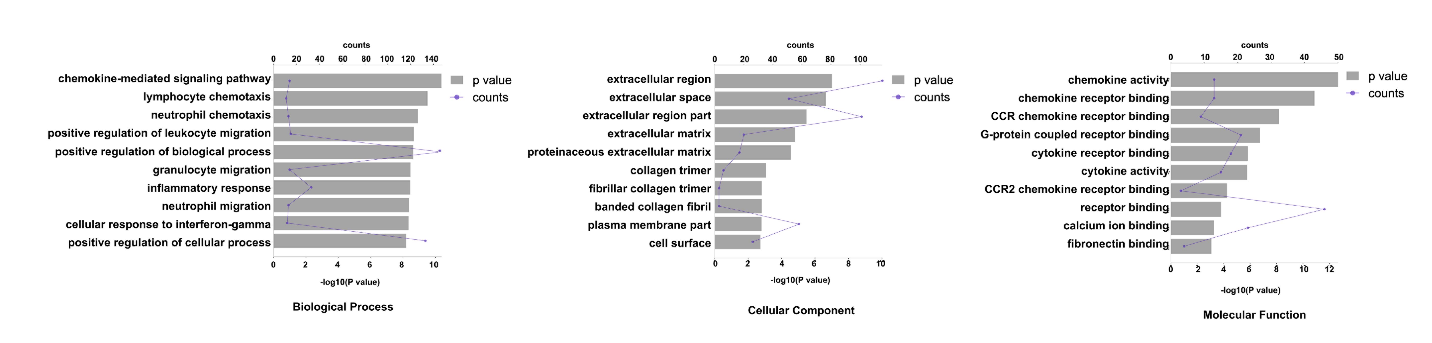


**Figure S7.** The top 10 pathways by GO enrichment analysis of the differentially expressed genes between aSC+iv.Tac_d_ and aSC+Col/Tac_5mg_ groups.


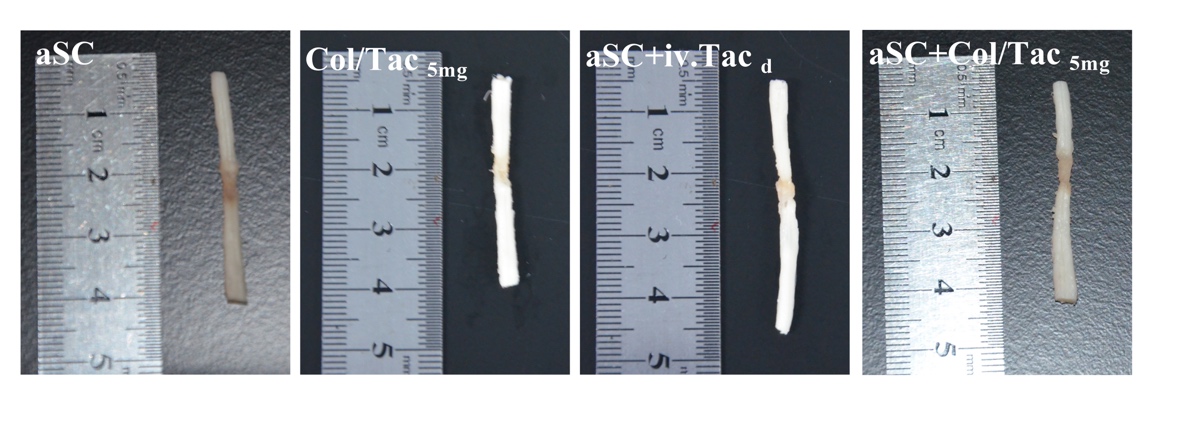


**Figure S8**. Photos of the spinal cord samples in the aSC, Col/Tac_5mg_, aSC+iv.Tac_d_ and aSC+ Col/Tac_5mg_ groups at 8-weeks post-surgery.


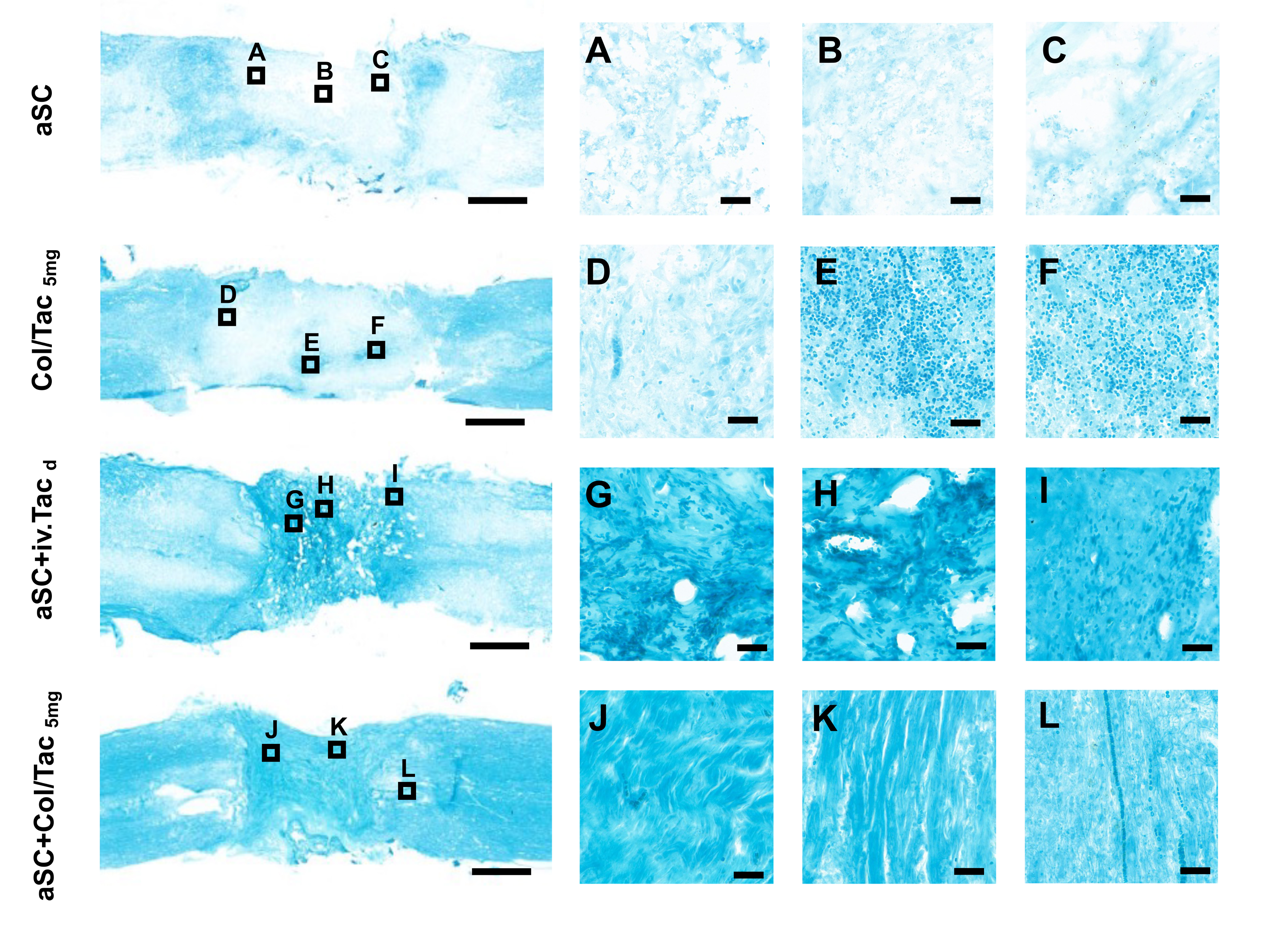


**Figure S9**. Myelin loss assessed by LFB staining at 60 days after aSC transplantation. Boxes (A-L) display enlarged fields of view of the spinal cord samples at lesion edge and epicenter. The Col/Tac treatment improved myelin sheath maintenance after aSC transplantation. Scale bars: 500 μm (left column), and 50 μm (A-L Boxes).
